# Phosphoproteome analysis reveals an extensive phosphorylation of proteins associated with bast fiber growth in ramie

**DOI:** 10.1186/s12870-021-03252-7

**Published:** 2021-10-16

**Authors:** Zheng Zeng, Fu Li, Renyan Huang, Yanzhou Wang, Touming Liu

**Affiliations:** 1 Institute of Bast Fiber Crops, Chinese Academy of Agricultural Sciences, Changsha, 410205 China; 2 Hunan Institute of Plant protection, Changsha, 410205 China

**Keywords:** Ramie, Differentially phosphorylated protein, Fiber growth, KNOX protein, Kinase, Phosphatase

## Abstract

**Background:**

Phosphorylation modification, one of the most common post-translational modifications of proteins, widely participates in the regulation of plant growth and development. Fibers extracted from the stem bark of ramie are important natural textile fibers; however, the role of phosphorylation modification in the growth of ramie fibers is largely unknown.

**Results:**

Here, we report a phosphoproteome analysis for the barks from the top and middle section of ramie stems, in which the fiber grows at different stages. A total of 10,320 phosphorylation sites from 9,170 unique phosphopeptides that were assigned to 3,506 proteins was identified, and 458 differentially phosphorylated sites from 323 proteins were detected in the fiber developmental barks. Twelve differentially phosphorylated proteins were the homologs of *Arabidopsis* fiber growth-related proteins. We further focused on the function of the differentially phosphorylated KNOX protein whole_GLEAN_10029667, and found that this protein dramatically repressed the fiber formation in *Arabidopsis*. Additionally, using a yeast two-hybridization assay, we identified a kinase and a phosphatase that interact with whole_GLEAN_10029667, indicating that they potentially target this KNOX protein to regulate its phosphorylation level.

**Conclusion:**

The finding of this study provided insights into the involvement of phosphorylation modification in ramie fiber growth, and our functional characterization of whole_GLEAN_10029667 provide the first evidence to indicate the involvement of phosphorylation modification in the regulation of KNOX protein function in plants.

**Supplementary Information:**

The online version contains supplementary material available at 10.1186/s12870-021-03252-7.

## Background

Post-translational modifications (PTMs) have been identified as playing important roles in diverse biological processes, including gene expression, cell signaling, protein stabilization, and the activation/deactivation of enzymatic activity [1]. At least 400 different types of PTMs have been detected in cells [1], indicating the considerable complexity of the PTM-mediated modulation of protein function. Protein phosphorylation modification is one of the most common types of PTMs, and widely participated in the growth and development of plants, including fiber formation [2]. Cellulose synthase genes (CesAs) are key genes associated with the synthesis of cellulose in fibers, and the functions of some CesA proteins, such as *Arabidopsis* AtCesA7 and CESA3, are known to be dynamically regulated by protein phosphorylation modification [3–5]. *PtMYB4* and *LTF1* are two fiber development-related MYB genes identified in *Pinus taeda* and *Populus*, respectively, and both of which can be phosphorylated by MAPK proteins [6, 7].

Ramie (*Boehmeria nivea* L. Gaud), also known as Chinese grass, is a fiber-producing crop, and has been cultivated for thousands of years in China [8, 9]. Ramie fibers, extracted from its stem bark, are among the most important raw materials in the production if industrial textiles. Although ramie fibers have numerous excellent characteristics, including long strands, smooth texture, and high tensile strength, they do have certain unfavorable traits, such as resistance to dyeing, confined elasticity and elongation potential, and the stiff brittle nature of the resultant cloth, which thus limit the quality of textiles produced from this material. Understanding for the developmental process of bast bark will benefit in improving ramie fiber yield and quality. Hence, over the past ten years, studies have focused on the identifying genes potentially involved in fiber development, based on homologous and/or expression analysis [10–14]. Furthermore, a large number of non-coding RNAs (ncRNAs) have been identified to play a potential role in the growth of ramie fiber [15, 16]. To date, however, there has been little evidence to indicate the involvement of PTMs in the fiber growth of this crop.

Plant fibers contain specialized secondary cellular walls that consist of primarily cellulose, hemicelluloses (xylan and glucomannan), and lignin [17], and thus the growth of fiber is mainly attributable to the secondary wall biosynthesis. It has been proposed that there is a specific snap point along the stem axis of fiber crops, which marks the transition from elongation to fiber thickening (above and below the snap point, respectively) [18]. Indeed, gene expression analysis has provided convincing evidence in support of the existence of snap points, characterized by a gradient of events associated with the shift from fiber elongation to thickening [19, 20]. Consistent with this supposition, microscopic examination of stem barks has revealed that bast fibers collected from different parts of stem barks are characterized by differences in developmental level, with barks in the top section of stems (TPS) showing no initiation of fiber growth, whereas barks in the middle section of stems (MPS) comprise a large number of fibers, the secondary walls of which are thickening (Figure S1) [12]. These observations would thus tend to indicate that the TPS and MPS lie above and below the snap point, respectively. To examine the role of protein phosphorylation modification in the growth of ramie fiber, we characterized and compared the phosphoproteome of TPS and MPS barks. We believe that the findings of this study will provide an important basis for understanding the involvement of PTMs in the regulation of fiber growth in ramie.

## Methods

### Experimental material, tissue sampling, and protein extraction

The Zhongzhu 1 used in this study is one of the extensively grown ramie cultivars in China. Seedlings of Zhongzhu 1 were grown at the experimental farm of the Institute of Bast Fiber Crops, Yuanjiang, China in June 2016. The TPS and MPS barks were separately sampled from five 30-day-old Zhongzhu 1 plants and immediately frozen in liquid nitrogen (Figure S1). For each bark type, three replicates were sampled. Sample of powdered bark obtained by grinding in liquid nitrogen was sonicated in lysis buffer, and then transferred to a 5-mL centrifuge tube, and sonicated three times on ice using a high intensity ultrasonic processor (Scientz) in lysis buffer (including 1% TritonX-100 (v/v), 10 mM dithiothreitol, and 1% protease inhibitor cocktail (v/v), 50 μM PR-619, 2 mM EDTA). An equal volume of Tris-saturated phenol (pH 8.0) was added. Then, the mixture was further vortexed for 5 min. After centrifugation (4 °C, 10 min, 5 000g), the upper phenol phase was transferred to a new centrifuge tube. Proteins were precipitated by adding at least four volumes of ammonium sulfate-saturated methanol and incubated at -20 °C for at least 6 h. After centrifugation at 4 °C for 10 min, the supernatant was discarded. The remaining precipitate was washed with ice-cold methanol, followed by ice-cold acetone for three times. Finally, the protein was redissolved in 8 M urea and the protein concentration was determined with BCA kit according to the manufacturer’s instructions.

### Fractionation and enrichment

The trypsin was used for proteins digestion, according to the description of Ye et al. [21]. The tryptic peptides of each sample were labeled by tandem mass tags (TMT) using the TMT kit according to the manufacturer’s protocol. Briefly, peptides were desalted by Strata X C18 SPE column (Phenomenex) and vacuum-dried. Peptide was reconstituted in 0.5 M TEAB and processed according to the manufacturer’s protocol for TMT kit/iTRAQ kit. The peptide mixtures were then incubated for 2 h at room temperature and pooled, desalted and dried by vacuum centrifugation. Then, sample was fractionated into fractions by high pH reverse-phase HPLC using Agilent 300 Extend C18 column (5 μm particles, 4.6 mm ID, 250 mm length). Briefly, peptides were separated with a gradient of 2% to 60% acetonitrile (v/v) in 10 mM ammonium bicarbonate pH 10 over 80 min into 80 fractions. Then, the peptides were combined into18 (proteome) / 6 (phosphoproteome) fractions and dried by vacuum centrifuging. Peptide mixtures were first incubated with IMAC microspheres suspension with vibration in loading buffer (50% acetonitrile/0.5% acetic acid (v/v). To remove the non-specifically adsorbed peptides, the IMAC microspheres were washed with 50% acetonitrile/0.5% acetic acid (v/v) and 30% acetonitrile/0.1% trifluoroacetic acid (v/v), sequentially. To elute the enriched phosphopeptides, the elution buffer containing 10% NH4OH (m/v) was added and the enriched phosphopeptides were eluted with vibration. The supernatant containing phosphopeptides was collected and lyophilized for LC-MS/MS analysis.

### UHPLC-MS/MS analysis

An LC-MS/MS system was used to perform phosphoproteome analysis of the enriched peptides. In brief, peptides were separated by dissolving in 0.1% formic acid (v/v, solvent A) using an EASY-nLC 1000 UPLC system. After separation, peptides were subjected to NSI source followed by tandem mass spectrometry (MS/MS) in a Q Exactive^TM^ Plus mass spectrometer (Thermo) coupled online to the UPLC system, operating with the parameters as followers: scan range, 350 to 1800 m/z; resolution, 70,000. Selection of peptides for MS/MS was carried out using NCE with a resolution of 17,500. Alternation between a single MS scan and 20 MS/MS scans was set for the data-dependent procedure, with a 15.0 s dynamic exclusion. The parameters of the fixed first mass and the automatic gain control was 100 m/z and 5E4, respectively .

### Database search and data analysis

The MS/MS data generated from phosphoproteome analysis were processed using MaxQuant search engine (v.1.5.2.8) [22]. Tandem mass spectra were searched against those obtained for protein sequences annotated from the genome of ramie [23], with the following parameters: only tryptic peptides with up to two missed cleavage sites permitted; 20 ppm (initial search) and 5 ppm (main search) mass tolerances for MS and 0.6 Da for MS/MS fragment ions; carbamidomethyl on cysteine as a fixed modification; and protein *N*-acetylation, oxidized methionine, and phospho-STY (serine, threonine, and tyrosine) permitted as variable modifications [21]. The false discovery rate was set to less than 1%, and only proteins with at least two peptides, among which at least one peptide was unique, were retained for further analysis.

The abundance of normalized phosphorylated proteins was determined based on the following two steps. We initially used the abundance of spike-in samples (quantified by TMT) to adjust the abundance among the three replicates, and thereafter, each TMT quantification channel within an individual replicate was normalized based on the total reporter ion intensity. Pearson correlation analysis was performed to evaluate the reproducibility of the three replicates. Fold change in the phosphorylation level of different protein sites was estimated by calculating the ratio of the normalized abundance of peptides underwent phosphorylated modification between two investigated samples. A protein site with a phosphorylation change greater than 2-fold was defined as a significantly differential site (*P* < 0.001).

### Parallel reaction monitoring analysis

Nine phosphorylated sites from six proteins were targeted for quantification via parallel reaction monitoring (PRM) analysis to verify the differences in phosphoproteomes detected based on UHPLC-MS/MS analysis. The methods used for protein extraction, trypsin digestion, and enrichment of phosphorylated peptides were the same as those used for phosphoproteome analysis. The peptide mixture was loaded onto a PicoFrit capillary column (Woburn, MA, USA) packed with ReproSil-Pur Basic C18 reverse-phase resin and separated using an EASY-nLC 1000 UPLC system, with a solvent B [0.1% formic acid (v/v) in 98% ACN (v/v)] gradient of 6% to 23% over 38 min, followed by 23% to 35% over 14 min, and then increasing to 80% over 4 min, with a flow rate of 400 nL/min. The eluate was examined via mass spectrometry using a Q Exactive^TM^ Plus mass spectrometer (Thermo) coupled to the UPLC system online. After a full-scan event, MS/MS scans in the PRM mode were triggered for target proteins. The set parameters were the same as those used for phosphoproteome UHPLC-MS/MS analysis. Three biological replicates were used. The PRM data thus obtained were analyzed using Skyline software with the following parameters: enzyme, trypsin (KR/P); max missed cleavages, 0; peptide length, 7-25 amino acids; static modification, Cys carbamidomethyl; variable modification, Met oxidation; max variable modifications, 3. The following transition settings were used: (1) precursor charges, 2 and 3; (2) ion charges, 1 and 2; (3) ion types, b, y and p; (4) product ions, from ion 3 to the final ion; and (5) ion match tolerance of 0.02 Da.

### Bioinformatic analysis

The functions of proteins identified based on phosphoproteome analysis were annotated by searching against the following public databases: InterPro, Gene Ontology (GO), and Kyoto Encyclopedia of Genes and Genomes (KEGG), with default parameter. Prediction of the subcellular localization of proteins was preformed using wolfpsort software [24]. Enrichment analysis of differentially phosphorylated proteins was performed using GOseq, by the Wallenius non-central hypergeometric distribution. Kinase prediction was carried out based a protein-protein interaction analysis by searching proteins against the STRING database [25]. Only interactions between the proteins within the searched data set were retained, thereby filtering external candidates. Protein pairs with a confidence score greater than 0.7 were defined as showing an interaction. Finally, we screened for kinases that interact with the candidate.

Orthologous analysis was performed for differentially phosphorylated proteins and known secondary wall-biosynthetic proteins of *Arabidopsis*, based on the method of bidirectional best hit (BBH) [26]. Sequence alignment between the ramie KONX protein whole_GLEAN_10029667 and *Arabidopsis* KNOX proteins was performed using the Clustal program [27], and then, an unrooted phylogenetic tree was constructed using MEGA 5 software based on the Neighbor-Joining method with 1,000 bootstrap replicates [28].

### Real-time quantitative PCR (qRT-PCR) analysis

To perform an expression analysis of three kinase genes, TPS and MPS barks (sites sampled as shown in Figure S1) were collected from 30-day-old Zhongzhu 1 plants, each with four replicates. Extraction of total RNA from each sample was carried out using the E.Z.N.A. Plant RNA kit. and After treating with DNase I (Fermentas, Canada), these isolated RNAs were used to generate first-strand cDNAs by reverse-transcribing using M-MuLV Reverse Transcriptase (Fermentas). Then, qRT-PCR was performed in optical 96-well plates using iTaq^TM^ Universal SYBR Green supermix (Bio-RAD, USA) in conjunction with an iQ5 multicolor real-time PCR system (Bio-RAD). The 18S ribosomal RNA gene was used as an internal control, and its primers sequences together with those of genes used for amplification are listed in Table S1. Relative expression levels were determined as previously described [29].

### Overexpression of whole_GLEAN_10029667

High-fidelity thermostable DNA polymerase was used to amplify the full-length cDNA of *whole_GLEAN_10029667* using the primer pair of Table S1. Then, the amplicon was ligated into the downstream of the CaMV 35S promoter in a PBI121 vector. Heat shock was carried out to introduced the constructed plasmid into *Agrobacterium tumefaciens* strain GV3101. Then, the floral dip method was used to infiltrate the transformed *Agrobacterium* into *Arabidopsis* [30]. Transgenic *Arabidopsis* plants were cultivated under the following conditions: environmental temperature, 22°C; light cycle ,16-h light and 8-h dark. Tissue sections of stem from the 40-day-old transgenic plants were stained using Safranin O-Fast Green, and then was observed under a transmission light microscope.

### Library construction for yeast two-hybridization assay

All tissues from seedlings, plant during the rapidly growing stage, and mature plants were collected and mixed for extraction of total RNA. The RNAs were further used to construct a cDNA library for the yeast two-hybridization assay, using a CloneMiner II cDNA Library Construction Kit (Invitrogen). Briefly, the SuperScript III First-Strand Synthesis System (Invitrogen) was used to synthesize the first-strand cDNA, and then amplified to obtain the double-stranded cDNAs (dscDNAs). Subsequently, BP recombination introduced the dscDNAs that had added attB1 adapters into pDONR 222 vector, thereby to generate a gateway entry library. Then, these resulting constructs were subjected to electrotransform into competent cells of *Escherichia coli* DH10B, and were incubated on the Luria-Bertani agar plate with 50 μg/ml kanamycin, thereby generating a primary cDNA library. Thereafter, after performing a DNA extraction for the primary cDNA library, the resulting plasmid DNA of was introduced into the pGADT7-DEST AD vector based on LR recombination, and finally to yield a secondary library. To evaluate the quality of the library, 24 colonies selected randomly from the cDNA library were used for amplification, and a total of 1.3 × 10^7^ cfu were included in the library based on capacity estimation.

### Yeast two-hybridization assay

To perform the yeast two-hybridization (Y2H) assay, we constructed the bait plasmid pGBKT7-*whole_GLEAN_10029667* (BD-*whole_GLEAN_10029667*) by inserting the full-length coding sequence of *whole_GLEAN_10029667* into a pGBKT7 vector. Subsequently, we used the Matchmaker Two Hybrid system (Clontech) to execute a Y2H screening. In brief, co-transformation of the BD-*whole_GLEAN_10029667* (bait) and cDNA library plasmids (prey) into Y2H competent yeast cells were carried out. Additionally, the positive and negative controls were set by co-transforming pGBKT7-53 plus pGADT7 and pGBKT7-Lam plus pGADT7 into competent yeast cells, respectively. Then, these co-transformants were incubated for 3–5 days on the SD/-Leu/-Trp/X-α-Gal agar plate. The generating blue colonies were selected for further validating on the higher stringency SD/-Leu/-Trp/-His/-Ade/-Aba/X-α-Gal agar plate.

Plasmid DNA was extracted from all blue colonies and sequenced to identify the interacting genes. To validate the interaction between whole_GLEAN_10029667 and its interacting candidate genes, the full-length coding sequences of the candidate genes were individually inserted into a pGADT7 vector, and these, along with BD-*whole_GLEAN_10029667*, were co-transformed into Y2H competent yeast cells. The empty pGADT7 vector was used as a control. These co-transformants were grown on SD/-Leu/-Trp/X-α-Gal and SD/-Leu/-Trp/-His/-Ade/-Aba/X-α-Gal agar plates, respectively.

## Results

### Characterization of the phosphoproteome of stem barks

**Fig. 1:**
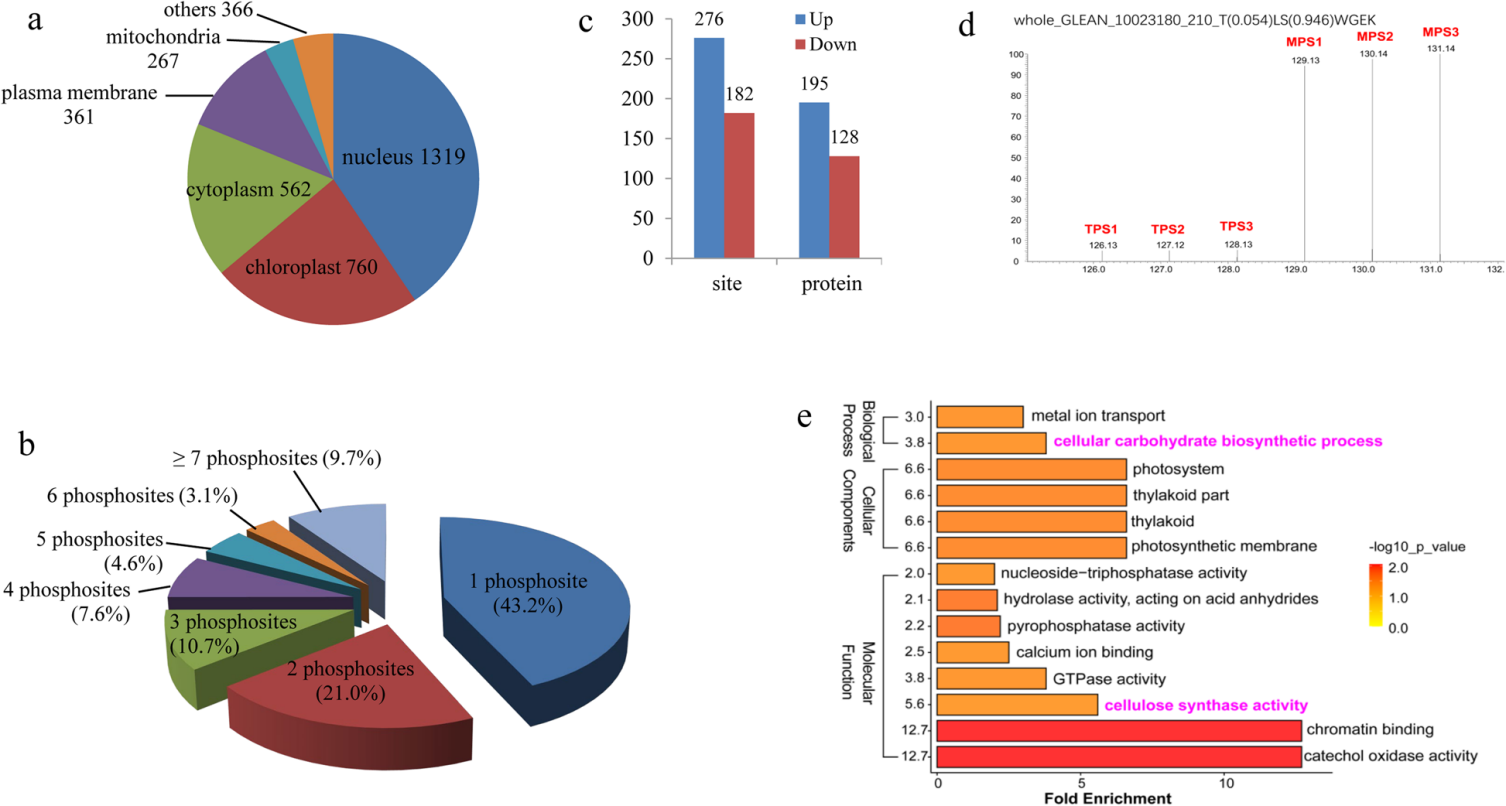
Characteristics of the phosphoproteomes of ramie bast barks. **a** Subcellular localizations of all 3,506 detected phosphoproteins based on GO annotation predictions. The numbers in the graph represent the number of proteins located in the corresponding organelles. **b** Proportion of single and multiple phosphorylation sites per protein in the phosphoproteome. **c** Number of differentially phosphorylated sites and proteins identified in the phosphoproteome of barks from the middle section of stems (MPS), compared with those in the top section of stems (TPS). **d** The secondary MS/MS spectra of the 210^th^ pSer site of whole_GLEAN_10023180 in the TPS and MPS barks. **e** GO terms enriched with proteins with down-regulated phosphorylation levels in MPS bark. GO terms significantly enriched for the three GO categories, Biological process, Cellular component, and Molecular function. The color of the circle represents the significance of the enrichment (*P* < 0.01)

We carried out phosphoproteomic analyses for the TPS and MPS of ramie bast barks using a UHPLC-MS/MS system, and accordingly obtained 154,142 spectra (Table 1). Subsequent analysis of these spectra enabled us to identify 9,170 unique phosphopeptides, which were assigned to 3,506 proteins with 10,320 detected phosphorylation sites (Table 1). To gain an overview of the functions of proteins identified from the phosphoproteome, we carried out a prediction of their subcellular localization based on GO annotation, and found that ~81.2% of the proteins were distributed in the chloroplast, cytoplasm, and/or nucleus (Fig. 1a). Notably, ~40.5% of these proteins were found to be distributed in the nucleus, indicating that a large number of phosphorylated proteins are potentially involved in the nuclear activity-related functions (including 195 for transcriptional regulation) in the bast barks of ramie.

**Table 1:** Summary of phosphoproteome analysis in the stem barks of ramie

Item	Number
Total spectrum	154,142
Peptides	9,170
Proteins	3,506
Phosphorylation sites	10,320
Differential proteins	323
Differential sites	458

The number of modification sites differed considerably among the identified phosphorylated proteins. Although we found that ~75.0% of the phosphoproteins had no more than three modification sites, 19 proteins in the phosphoproteome were identified as having more than 20 such sites (Fig. 1b), whereas whole_GLEAN_10023576, a cell surface glycoprotein, was found to have 39 phosphorylation sites. Among the 10,320 identified phosphorylation sites, 85.9% (8,869) were phosphorylated on serine (pSer), 13.7% (1,410) on threonine (pThr), and only 0.4% (41) on tyrosine (pTyr).

### Differentially phosphorylated proteins in the barks of TPS and MPS

Our observation of good repeatability in parallel replicates from the datasets obtained in this study (Figure S2) indicated that these datasets were suitable for further use in detecting the differential phosphorylation of proteins in the two investigated bast barks. Quantification of the phosphorylated protein revealed that at the set threshold (2.0), 276 (60.3%) sites in 195 proteins were deemed to be up-regulated targets in the MPS, whereas 182 (39.7%) sites in 128 proteins were quantified as down-regulated targets (Fig. 1c). Of these differentially phosphorylated proteins, five sites from three proteins showed a greater than 9-fold increase (Table S2), notable among which was the 210^th^ Ser-site of whole_GLEAN_10023180, at which the phosphorylation level was found to have increased by 15.6-fold in the fiber-growing bark (Fig. 1d). The whole_GLEAN_10023180 is a type of kinesin protein, and in *Arabidopsis*, kinesin plays important roles in fiber formation by mediating the deposition of secondary walls [31, 32].

To gain an overview of the proteins with different phosphorylation status, we performed GO enrichment analysis , and accordingly identified a total of 14 GO terms that were significantly enriched with proteins characterized by down-regulated phosphorylation, including “cellulose synthase activity” and “cellular carbohydrate biosynthetic process” (Fig. 1e, Table S3). It is known that secondary wall biosynthesis of fibers primarily involves cellular carbohydrate metabolism, such as cellulose biosynthesis catalyzed by cellulose synthase, and thus our observations may imply that modification via phosphorylation plays a role in regulating fiber growth.

### Identification of candidates for fiber growth among the differentially phosphorylated proteins

The growth of plant fibers mainly reflects the biosynthesis and deposition of secondary walls. To identify candidate proteins associated with fiber growth, we performed an orthologous analysis between differentially phosphorylated proteins and known secondary wall biosynthetic proteins of *Arabidopsis*. Our results revealed a total of 12 orthologs of *Arabidopsis* known secondary wall biosynthetic proteins, containing 29 sites showing considerable differences in phosphorylation status between the two investigated types of bark tissues (Table 2). Of these 12 proteins, there were three CesAs, one homolog of endo-1,4-beta-glucanase KOR [33], and one homolog of xylosyltransferase IRX9 [34].

**Table 2:** Differentially phosphorylated sites from 12 proteins that are the homolog of Arabidopsis fiber growth-related proteins

Protein ID	Position	Normalized abundance		Change fold (MPS/TPS)	Direction	P value	Amino acid	CP (%)	Modified sequence	Annotation
		MPS	TPS							
whole_GLEAN_10007243	190	1.53	0.47	3.30	Up	4.07E-05	S	100	VHPYPVS(ph)EHGSAR	Cellulose synthase IRX3
	194	1.56	0.44	3.57	Up	1.95E-05	S	100	VHPYPVSEHGS(ph)AR	
whole_GLEAN_10016752	241	0.42	1.58	0.27	Down	5.72E-05	T	94.1	GGDIDAST(ph)DVLVDDSLLNDEAR	Cellulose synthase
	198	0.32	1.68	0.19	Down	5.50E-05	S	100	VGDPVRDFGS(ph)PGLGNVAWK	
	163	0.57	1.43	0.40	Down	2.07E-05	S	100	FSMAS(ph)PGVGGAK	
	92	0.31	1.69	0.18	Down	5.53E-05	S	100	GSPAILGDKEEDDVDDGAS(ph)DFNYPSENQNEK	
	117	0.62	1.37	0.45	Down	1.99E-05	T	93.9	MLSWQMT(ph)YGR	
	113	0.50	1.50	0.33	Down	1.50E-05	S	100	MLS(ph)WQMTYGR	
whole_GLEAN_10027761	147	0.53	1.47	0.36	Down	1.98E-05	S	89.7	TTS(ph)GPLGPGDK	Cellulose synthase
	111	0.63	1.37	0.46	Down	1.63E-04	S	82.0	GEDADLSSS(ph)SR	
	110	0.43	1.57	0.28	Down	2.23E-05	S	78.4	GEDADLSS(ph)SSR	
whole_GLEAN_10013375	141	1.42	0.61	2.35	Up	6.63E-04	S	81.1	RGDS(ph)SSSVSYR	Exocyst complex component EXO70A1
whole_GLEAN_10011928	38	0.47	1.53	0.31	Down	4.82E-04	S	85.3	AALSS(ph)RPLDETQQSWLLGPAMEK	Endo-1,4-beta-glucanase KOR
	37	0.56	1.44	0.39	Down	3.70E-05	S	85.4	AALS(ph)SRPLDETQQSWLLGPAMEK	
whole_GLEAN_10022707	630	1.44	0.69	2.10	Up	2.88E-03	S	100	GGPNEKDS(ph)NNDNASVR	Xylosyltransferase IRX9
whole_GLEAN_10015497	140	0.60	1.54	0.39	Down	1.20E-04	S	100	GSGGDFS(ph)GDK	MYB protein
whole_GLEAN_10022328	482	1.64	0.36	4.55	Up	1.19E-04	S	96.3	EKS(ph)PGSNTNTNTDNQTASFTPK	BEL1-like homeodomain protein
	499	1.44	0.56	2.58	Up	5.68E-05	T	81.6	EKSPGSNTNTNTDNQTASFT(ph)PK	
	229	1.35	0.65	2.08	Up	9.23E-04	S	99.3	AVGESSGAAAS(ph)GEGSGAVGSGGGGEGGSGK	
	485	1.44	0.56	2.58	Up	5.68E-05	S	89.3	EKSPGS(ph)NTNTNTDNQTASFTPK	
whole_GLEAN_10029667	261	1.62	0.38	4.31	Up	5.83E-05	S	100	KYS(ph)GYLSGLK	Knotted-like homeobox protein
whole_GLEAN_10020389	73	0.55	1.45	0.38	Down	8.24E-05	S	100	GRS(ph)FDDAEPYISNNK	kinesin-13A
whole_GLEAN_10023180	189	1.78	0.19	9.26	Up	1.41E-04	S	100	PAS(ph)FKPFVR	kinesin protein FRA1
	795	1.54	0.45	3.42	Up	9.80E-05	S	100	S(ph)LGELPSGR	
	775	1.69	0.30	5.75	Up	4.14E-03	S	99.6	SSS(ph)PDRFR	
	210	1.84	0.12	15.58	Up	5.75E-05	S	94.6	TLS(ph)WGEK	
	198	1.48	0.51	2.88	Up	9.63E-04	S	96.3	KNS(ph)EPFANSFLR	
	771	1.49	0.51	2.95	Up	1.26E-03	S	100	EHLQYS(ph)R	
whole_GLEAN_10023749	1579	1.64	0.38	4.33	Up	1.84E-05	S	99.8	RAS(ph)FGGSVAYEPCLHAK	phosphoinositide phosphatase SAC1

*Note*: TPS and MPS indicated the bark sample collected from the top and middle part of stems; CP represented confidence percentage for phosphorylation modification site

EXO70A1 is a subunit of the exocyst complex involved in the targeted transport of CesA complexes [35], whereas FRA1 and Kinesin-13A are two kinesin proteins required for cellulose microfibril deposition and microtubule depolymerization, respectively [31, 32] and SAC1 is a phosphoinositide phosphatase that is essential for actin organization and secondary wall deposition [36]. In our analysis of MPS bark (characterized by a thickening of the secondary walls of fibers), we detected distinct increases in phosphorylation levels of the ramie homologs of FRA1, SAC1, and EXO70A1, whereas in contrast, a reduction in levels was observed in the Kinesin-13A-homologous whole_GLEAN_10020389 (Table 2). In addition, the phosphorylation levels of three transcriptional regulators (one MYB protein, one BEL1-like homeodomain protein, and one KNOX protein) were found to show significant changes during the fiber developmental phase of bark (Table 2).

Taken together, our observations provide evidence to indicate that these 12 proteins play roles in the secondary wall biosynthesis of ramie fibers, and that their functions are probably regulated by phosphorylation modification.

### Validating the difference in candidate proteins

**Fig. 2:**
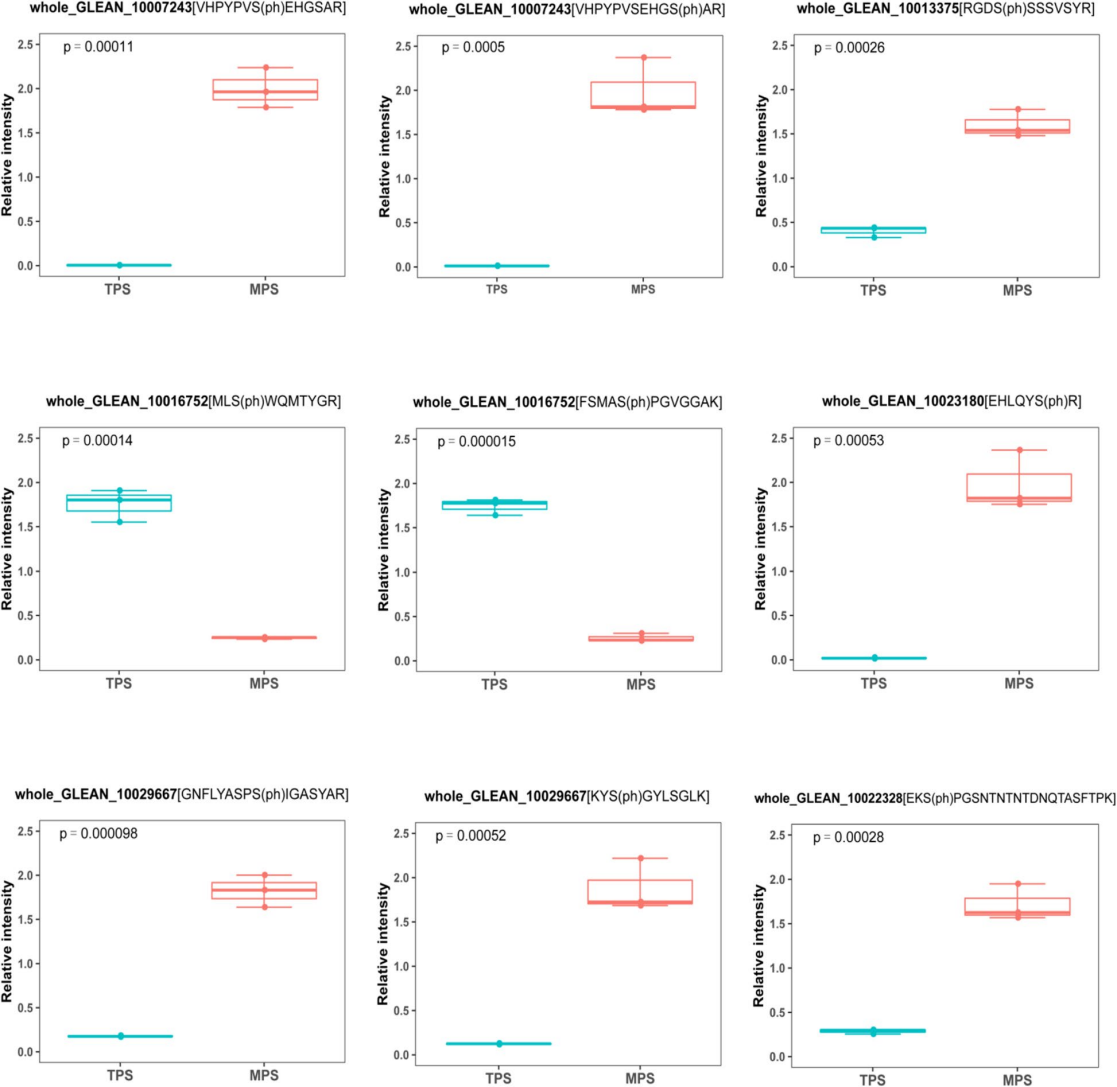
Validation of the phosphorylated change at nine sites from six proteins homologous to *Arabidopsis* fiber growth-related proteins

Parallel reaction monitoring (PRM) technology has been confirmed to be a highly efficient tool for quantifying the phosphorylation level of target proteins [37], and we accordingly performed PRM analysis to verify the differences at nine sites from six candidate proteins in the two investigated barks, namely, two CesAs (whole_GLEAN_10007243 and whole_GLEAN_10016752), the orthologs of FRA1 (whole_GLEAN_10023180) and EXO70A1 (whole_GLEAN_10013375), a BEL1-like homeodomain transcription factor (whole_GLEAN_10022328) and a KNOX transcription factor (whole_GLEAN_10029667). The results obtained were found to be consistent with those based on the phosphoproteome analysis (Fig. 2). Given the proteins analyzed using PRM technology were randomly selected, we can assume that the differences in candidate proteins identified based on phosphoproteome analysis are reliable.

### Prediction of kinases interacting with the candidate proteins

**Fig. 3:**
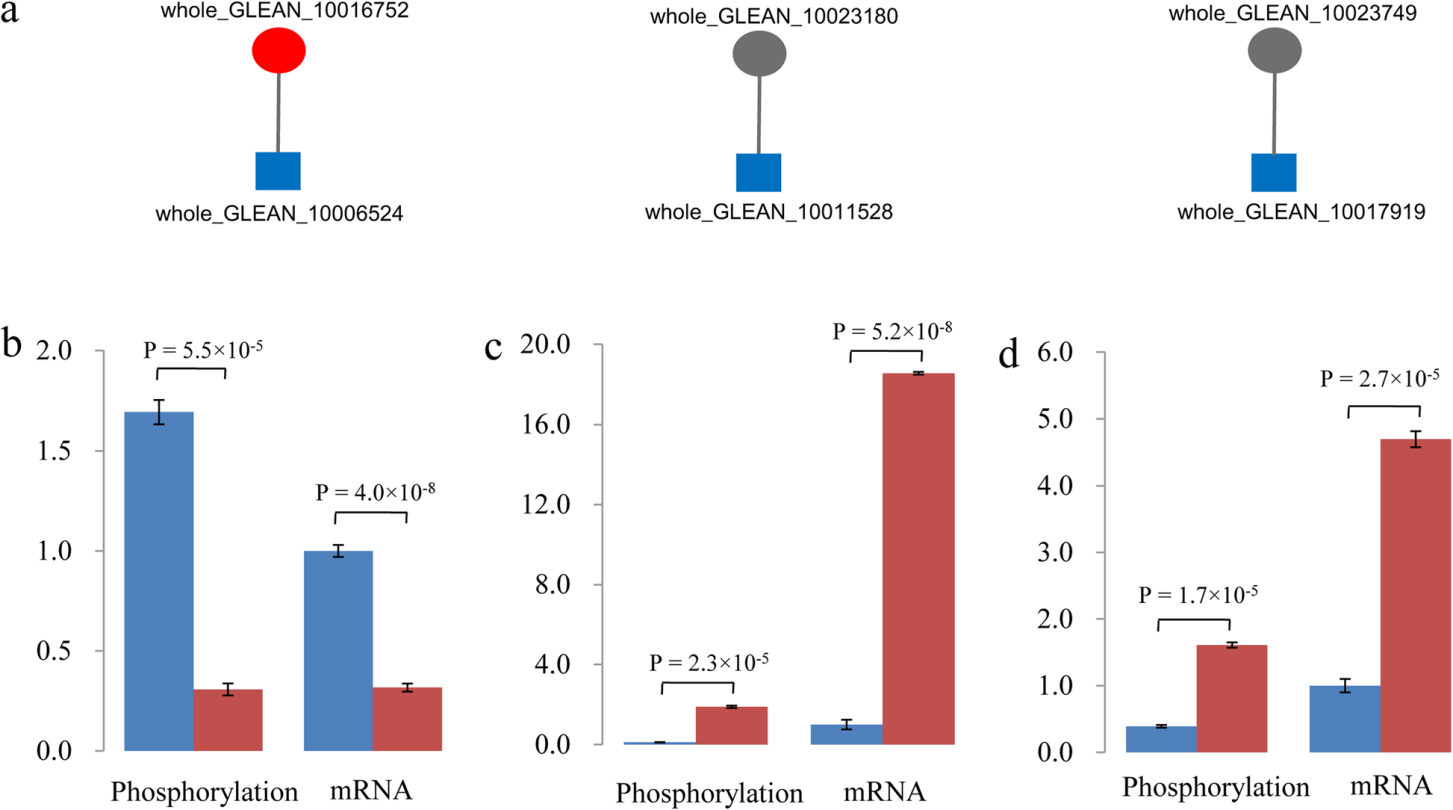
Identification of interacting kinases. **a** Kinases identified from the bioinformatic prediction for three fiber growth-related candidate proteins. The red and gray circles indicate the down- and up-regulated phosphorylation levels for the candidate proteins, respectively. The changes in phosphorylation levels of candidate proteins and mRNA expression of the corresponding kinase genes are shown in **b**. (whole_GLEAN_10016752 and kinase gene whole_GLEAN_10006524), **c**. (whole_GLEAN_10023180 and kinase gene whole_GLEAN_10011528), and **d**. (whole_GLEAN_10023749 and kinase gene whole_GLEAN_10017919). The blue and red columns represent the abundances observed in the barks from the top and middle sections of stems, respectively

To identify the putative kinases involved in phosphorylated modulation of the 12 identified fiber developmental candidate proteins, we performed protein-protein interaction predictions by searching against the STRING database [25]. We accordingly identified three kinases that putatively interact with the CesA protein whole_GLEAN_10016752, FRA1 homolog whole_GLEAN_10023180, and SAC1 homolog whole_GLEAN_10023749, respectively (Fig. 3a). In addition, qPCR analysis revealed significant changes in the expression of genes encoding these three kinases in the fiber-developmental bark. Notably, we observed a consistent pattern in the direction of regulation between changes in the expression of the three kinase genes and changes in the phosphorylation status of the corresponding target proteins in fiber-developmental bark, that is, in response to an up-regulated expression of kinases in the fiber-developmental bark, we observed an increase in the level of phosphorylation in the corresponding target protein, and vice versa (Fig. 3b-d). These expression results accordingly provide convincing evidence confirming the veracity of our kinase predictions.

### Functional characterization of the KNOX protein whole_GLEAN_10029667

**Fig. 4:**
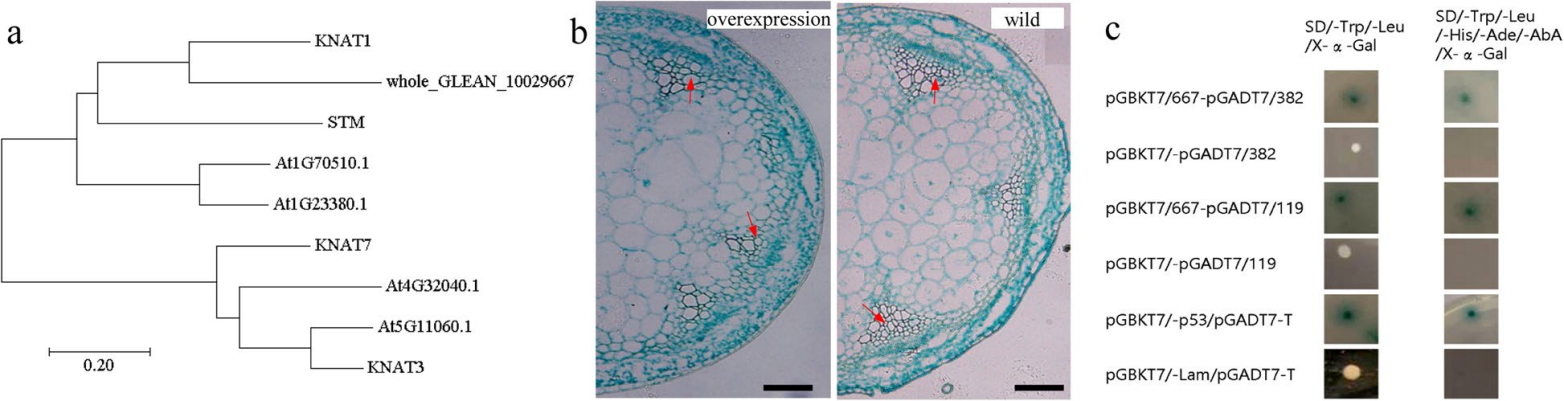
Functional characterization of the ramie KNOX protein whole_GLEAN_10029667. **a** Phylogenetic relationships among *Arabidopsis* KNOX proteins and whole_GLEAN_10029667. **b** Microscopic examination of transverse sections of the stems of whole_GLEAN_10029667-overexpressing (left) and wild-type *Arabidopsis* (right) plants, at ×100 magnification. The red arrows indicate fiber cells. The scale bar represents 200 μm. **c** Verification of interactions with whole_GLEAN_10029667 from a one-to-one Y2H assay. The left and right rows represent the yeast colonies cultivated on SD/-Leu/-Trp/X-α-Gal and SD/-Leu/-Trp/-His/-Ade/-Aba/X-α-Gal agar plates, repressively. The colonies in the first line are yeasts containing both pGBKT7-whole_GLEAN_10029667 (pGBKT7/667) and pGADT7- whole_GLEAN_10002382 (pGADT7/382) constructs, whereas colonies in the second line are those of control yeast transformed with an empty pGBKT7 vector. The colonies in the third line indicate the interaction of whole_GLEAN_10029667 and whole_GLEAN_10021119 by transferring both pGBKT7-whole_GLEAN_10029667 (pGBKT7/667) and pGADT7- whole_GLEAN_10021119 (pGADT7/119) constructs into yeast, whereas the colonies in the fourth line are those of the control into which an empty pGBKT7 vector was transferred. The fifth and sixth lines show the positive and negative controls used in the Y2H assay, respectively

The findings of numerous studies have provided evidence to indicate that KNOX genes play key roles in plant fiber growth [38–42]. In the present study, based on both the phosphoproteome and PRM analyses, we found that the KNOX protein whole_GLEAN_10029667 identified in the fiber-developmental bark of ramie showed a considerably higher level of phosphorylation compared with that in the TPS bark. Our sequence analysis revealed that whole_GLEAN_10029667 is an ortholog of KNAT1 (Fig. 4a), an important regulatory protein associated with fiber growth in *Arabidopsis*. We subsequently overexpressed *whole_GLEAN_10029667* in *Arabidopsis*, and thereby established that this ramie KNOX gene is associated with a marked reduction of fiber number in the stems of *Arabidopsi*s (Fig. 4b). Collectively, our results strongly imply that the ramie protein whole_GLEAN_10029667 functions as a negative regulator to inhibit the fiber growth of ramie, and its differential phosphorylation status in fiber-developmental barks indicate the function of this protein is probably mediated via phosphorylation modification.

**Fig. 5:**
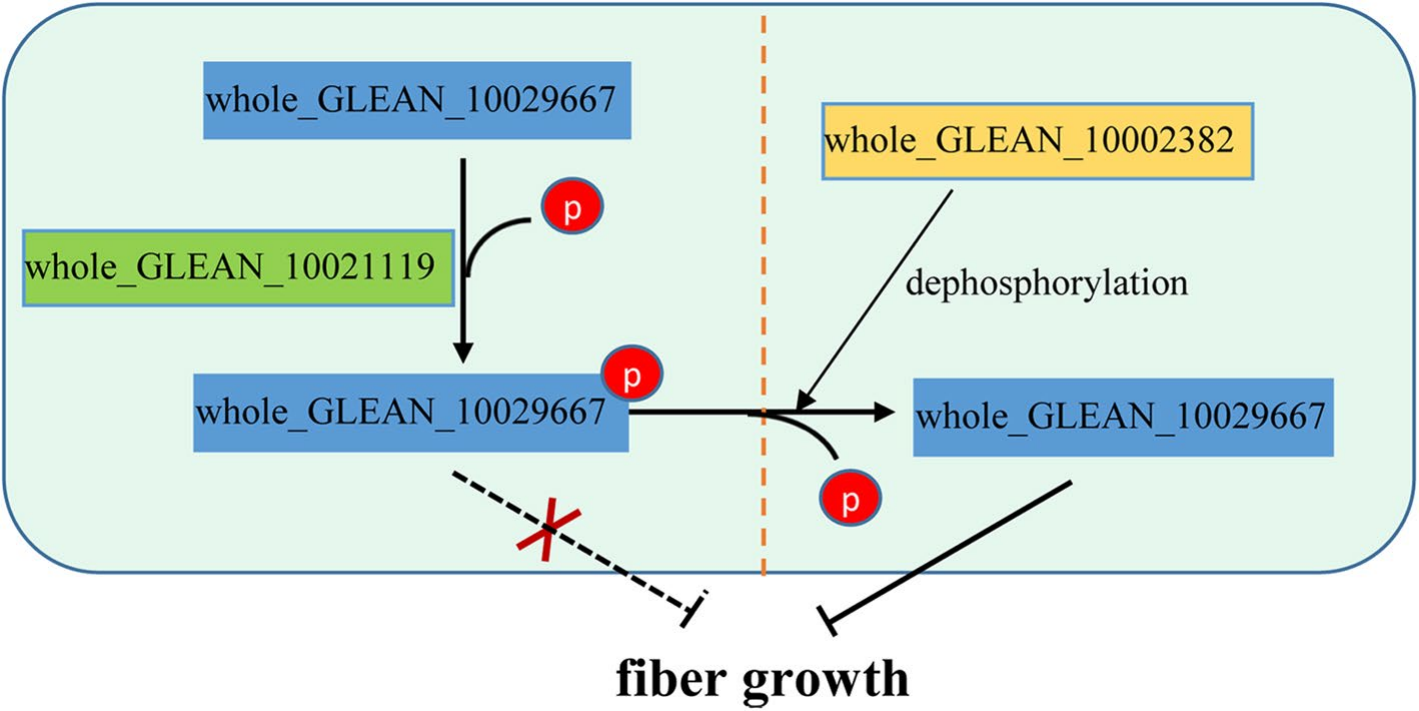
A putative model for the functional mechanisms wherebywhole_GLEAN_10029667 inhibits fiber growth in ramie. The kinase whole_GLEAN_10021119 and phosphatase whole_GLEAN_10002382 mediate the phosphorylation level of whole_GLEAN_10029667, by catalyzing its phosphorylation and dephosphorylation, respectively, thereby influencing the function of whole_GLEAN_10029667 in the inhibition of fiber growth

On the basis of bioinformatics protein-protein interaction analysis, we were unable to identify a kinase associated with whole_GLEAN_10029667, and consequently, in an effort to identify a kinase that targets whole_GLEAN_10029667, we constructed a yeast library of ramie cDNA, and then performed a Y2H assay. We accordingly succeeded in detecting a total of 15 proteins that interacted with whole_GLEAN_10029667 in the yeast system (Table 3), including four proteases and four transcriptional factors. Notably, we detected a kinase (whole_GLEAN_10021119) and a phosphatase (whole_GLEAN_10002382) that interact with the target KNOX protein, and are thus considered good candidates for mediating the observed changes in phosphorylation status of whole_GLEAN_10029667. Subsequently, we performed a one-to-one Y2H analysis between whole_GLEAN_10029667 and whole_GLEAN_10021119/ whole_GLEAN_10002382, which further verified their interactions (Fig. 4c). On the basis of this evidence, we propose the following model to account the functional mechanism of whole_GLEAN_10029667: (1) the KNOX protein whole_GLEAN_10029667 inhibits fiber growth in the fiber-developmental bark of ramie; (2) kinase whole_GLEAN_10021119 and phosphatase whole_GLEAN_10002382 mediate the level of whole_GLEAN_10029667 phosphorylation, by catalyzing its phosphorylation and dephosphorylation, respectively; (3) changes in phosphorylation level influence the function of whole_GLEAN_10029667 in the inhibition of fiber growth, thereby precisely controlling fiber growth in ramie; and (4) an increase in the level of whole_GLEAN_10029667 phosphorylation probably causes the disinhibition of fiber growth in the fiber-developmental bark of ramie, thereby leading to the normal growth of fibers (Fig. 5) .

**Table 3:** Candidate proteins interacted with KNOX protein whole_GLEAN_10029667 screened from the yeast double hybridization test

Interacted protein	Annotation
whole_GLEAN_10029792	Cysteine proteinase RD21a
whole_GLEAN_10022617	Thioredoxin-like 3-3
whole_GLEAN_10021424	MpBBI partial
whole_GLEAN_10021119	ribose-phosphate pyrophosphokinase
whole_GLEAN_10018956	lanyl-tRNA editing protein AlaX-L
whole_GLEAN_10016954	Agamous-like MADS-box protein AGL16
whole_GLEAN_10016497	ATP-dependent zinc metalloprotease FTSH 11
whole_GLEAN_10023005	ATP-dependent Clp protease
whole_GLEAN_10023094	probable aspartyl protease
whole_GLEAN_10011656	Transcription factor ORG2
whole_GLEAN_10011657	Transcription factor ORG2
whole_GLEAN_10011658	Transcription factor ORG2
whole_GLEAN_10002382	Dual specificity protein phosphatase
whole_GLEAN_10025202	1 2-dihydroxy-3-keto-5-methylthiopentene dioxygenase
whole_GLEAN_10023758	hypothetical protein L484_020387

### Discussion

The modification of protein via phosphorylation has been identified to play an important role in mediating the growth of fibers in plants. For example, the phosphorylation modification of fiber growth-related CesA and MYB proteins has been found to have a marked influence on their function [2, 6, 7]. Although ramie fibers currently have significantly economic importance in the textile industry, from the perspective of improving the fiber traits of this crop, it would be desirable to gain a more in-depth understanding of the mechanisms underlying fiber growth. To date, however, there has been comparatively little research devoted to examining the involvement of protein phosphorylation modification in the growth of ramie fibers. In this study, we detected 323 proteins that were either phosphorylated or dephosphorylated in the fiber-developmental bast bark of ramie, thereby providing the first evidence indicating the extensive phosphorylation of proteins associated with fiber growth. Notably, although we detected differences in the phosphorylation status of these 323 proteins in the two types of bark tissue investigated, all showed a certain extent of modified phosphorylation in both TPS and MPS, thereby providing evidence of the involvement of dynamic change in phosphorylation levels with respect to the fine regulation of protein function. Among the 323 differentially phosphorylated proteins, we identified 12 homologs of *Arabidopsis* fiber growth-related proteins, including the CesAs and MYB proteins, and the putative functional roles of which in fiber growth were inferred based on orthologous analysis. Specifically, we identified five proteins (three CesAs, one EXO70A1 homolog and one KOR homolog) and one (IRX9 homolog) that function as the enzymes in the catalysis of cellulose and hemicellulose biosynthesis; three (homologs of FRA1, SAC1 and Kinesin-13A) that are potentially involved in the deposition of secondary walls; and the other three that play roles in the transcriptional regulation of fiber growth.

In all examined plant lineages, KNOX proteins belonging to the THREE AMINO ACID LOOP EXTENSION (TALE) transcription factor family have been characterized as regulators of different aspects of development, and play key roles in maintaining a pluripotent cell population, referred to the shoot apical meristem, at the growing tips of seed plants [43]. Recently, KNOX proteins have also been shown to play a negative role in fiber growth by mediating reductions in the deposition of secondary cellular walls in *Arabidopsis* [38, 39, 41, 42]. However, among the class I KNOX protein of *Arabidopsis*, such as KNAT1 and STM, a loss of function results in a reduction in xylem fibers [40]. Accordingly, the findings of these previous studies provide evidence to indicate that KNOX proteins play potentially roles in the regulation of fiber growth. Notably, KNOX proteins can repress the expression of lignin biosynthetic genes, either directly by binding to the promoters of these genes or indirectly by forming heterodimers with BELL proteins, thereby negatively regulating lignin biosynthesis [44]. In the present study, we found that the ramie fiber development-regulated KNOX protein whole_GLEAN_10029667 underwent phosphorylated modification during the fiber growth stage, and identified an associated kinase (whole_GLEAN_10021119) and phosphatase (whole_GLEAN_10002382). These observations thus provide the first evidence for the involvement of phosphorylation modification in mediating the function for KNOX protein in plants. In this regard, it is known that ramie fibers have relatively low lignin content (~1.53%) when compared with the fibers of other crops, such as jute (~12.45%), kenaf (~8.8%), and hemp (~6.38%), which conceivably indicates that the fine functional regulation of whole_GLEAN_10021119 via phosphorylation modification plays a role in the formation of hypolignified fibers in ramie.

## Conclusion

This study identified 458 differentially phosphorylated sites from 323 proteins in the fiber developmental stem barks of ramie from the phosphoproteomic analysis. Of these proteins, 12 were the homolog of *Arabidopsis* fiber growth-related proteins. Overexpression of the differentially phosphorylated KNOX protein whole_GLEAN_10029667 revealed that this protein repressed fiber formation. Additionally, a kinase and a phosphatase showed an interaction with whole_GLEAN_10029667, suggesting that they targeted this KNOX protein to regulate its phosphorylation level; thereby we proposed a model for the functional mechanism of whole_GLEAN_10029667 in fiber growth.

## Supplementary Information

**Additional file 1: Table S1.** The sequences of primers used in this study.

**Additional file 2: Table S2.** Differentially Phosphorylated sites in the 323 differentially phosphorylated proteins identified in this study.

**Additional file 3: Table S3.** GO terms significantly enriched with proteins in which phosphorylation was up-regulated in fiber-developmental bark.

**Additional file 4: Figure S1.** Microscopic observation of fiber cells from the stem barks of ramie (published by Chen et al. 2014, BMC Genomics, 15:919). Barks collected from the top (TPS) and middle (MPS) sections of ramie stems were used for phosphoproteome analysis in this study. MPS is characterized by secondary cellular wall growth initiation and thickening, whereas the initiation of growth is not seen in TPS. Red arrows indicate the differential thickness of the cell walls of fibers from the two tissue types. Scale bars shown in the microscopic sections of TPS and MPS and in the figure of whole plant are 20 μm and 10 cm in length.

**Additional file 5: Figure S2.** Evaluation of the repeatability among replicate samples based on Pearson correlation analysis. The numbers in the graph indicate the correlation coefficient, and the red and blue table represent the positive and negative correlations, respectively. TPS and MPS indicate the phosphoproteome for the barks collected from the top and middle sections of stems, respectively.

## Data Availability

The mass spectrometry proteomics data have been deposited to the ProteomeXchange Consortium (http://proteomecentral.proteomexchange.org) via the iProX partner repository with the dataset identifier PXD024896.

## Abbreviations

PTM: Post-translational modification
CesA: Cellulose synthase genes
ncRNA: non-coding RNA
TPS: barks in the top part of stem
MPS: barks in the middle part of stem
TMT: tandem mass tags
PRM: parallel reaction monitoring analysis
qRT-PCR: Real-time quantitative PCR analysis
GO: Gene Ontology
Y2H: yeast double hybridization test
