# Supplementary material for: Phosphoproteome analysis reveals an extensive phosphorylation of proteins associated with bast fiber growth in ramie
Source: BMC Plant Biol. 2021 Oct 16;21:473. doi: 10.1186/s12870-021-03252-7 (PMC8520194; doi:10.1186/s12870-021-03252-7)
Supplement: Supplementary file 1 — Additional file 1: Table S1. The sequences of primers used in this study. [file 12870_2021_3252_MOESM1_ESM.docx]

**Table S1** The sequences of primers used in this study

| **Purpose** | | | **Sequence** | | |
| --- | --- | --- | --- | --- | --- |
| cDNA amplifying | *whole_GLEAN_10029667* | Forward primer | | ATGGAGGAATACAATAACATGGCCG |  |
|  |  | Reverse primer | | TTATGGGCCGAGACGGTAGT |  |
| Constructing overexpression vector | *whole_GLEAN_10029667* | Forward primer | | CACGGGGGACTCTAGAGGATCCATGGAGGAATACAATAACATGGCCG |  |
|  |  | Reverse primer | | GGGACTGACCACCCGGGGATCCTTATGGGCCGAGACGGTAGT |  |
| qRT-PCR | *whole_GLEAN_10006524* | Forward primer | | ACTGGGTTCGCCTCTACTT |  |
|  |  | Reverse primer | | GTTCGATGGGATGAATGTGAG |  |
|  | *whole_GLEAN_10011528* | Forward primer | | CAGGGACAGGTCATTGTGATA |  |
|  |  | Reverse primer | | TCTGAGGTTTGGAAGAAAGC |  |
|  | *whole_GLEAN_10017919* | Forward primer | | AAGATTCTGTGCGTCGGTT |  |
|  |  | Reverse primer | | ACATTGTGAGACCCATAGACC |  |
|  | *18s* | Forward primer | | AGACTGTGAAACTGCGAATG |  |
|  |  | Reverse primer | | AATCATCTGAGCAACGGG |  |
