# Supplementary material for: Phosphoproteome analysis reveals an extensive phosphorylation of proteins associated with bast fiber growth in ramie
Source: BMC Plant Biol. 2021 Oct 16;21:473. doi: 10.1186/s12870-021-03252-7 (PMC8520194; doi:10.1186/s12870-021-03252-7)
Supplement: Supplementary file 3 — Additional file 3: Table S3. GO terms significantly enriched with proteins in which phosphorylation was up-regulated in fiber-developmental bark. [file 12870_2021_3252_MOESM3_ESM.docx]

**Table S3** GO terms significantly enriched with proteins in which phosphorylation was up-regulated in fiber-developmental bark

|  | GO Terms | Description | Fold | P value |
| --- | --- | --- | --- | --- |
| Cellular Component | GO:0009521 | photosystem | 6.6 | 3.2×10^-2^ |
|  | GO:0044436 | thylakoid part | 6.6 | 3.2×10^-2^ |
|  | GO:0009579 | thylakoid | 6.6 | 3.2×10^-2^ |
|  | GO:0034357 | photosynthetic membrane | 6.6 | 3.2×10^-2^ |
| Molecular Function | GO:0003682 | chromatin binding | 12.7 | 8.8×10^-3^ |
|  | GO:0004097 | catechol oxidase activity | 12.7 | 8.8×10^-3^ |
|  | GO:0016462 | pyrophosphatase activity | 2.2 | 2.1×10^-2^ |
|  | GO:0016817 | hydrolase activity, acting on acid anhydrides | 2.1 | 2.2×10^-2^ |
|  | GO:0003924 | GTPase activity | 3.8 | 4.2×10^-2^ |
|  | GO:0017111 | nucleoside-triphosphatase activity | 2.0 | 4.4×10^-2^ |
|  | GO:0005509 | calcium ion binding | 2.5 | 4.4×10^-2^ |
|  | GO:0016759 | cellulose synthase activity | 5.6 | 4.6×10^-2^ |
|  |  |  |  |  |
| Biological Process | GO:0030001 | metal ion transport | 3.0 | 4.1×10^-2^ |
|  | GO:0034637 | cellular carbohydrate biosynthetic process | 3.8 | 4.2×10^-2^ |
