# Supplementary material for: Phosphoproteome analysis reveals an extensive phosphorylation of proteins associated with bast fiber growth in ramie
Source: BMC Plant Biol. 2021 Oct 16;21:473. doi: 10.1186/s12870-021-03252-7 (PMC8520194; doi:10.1186/s12870-021-03252-7)
Supplement: Supplementary file 4 — Additional file 4: Figure S1. Microscopic observation of fiber cells from the stem barks of ramie (published by Chen et al. 2014, BMC Genomics, 15:919). Barks collected from the top (TPS) and middle (MPS) sections of ramie stems were used for phosphoproteome analysis in this study. MPS is characterized by secondary cellular wall growth initiation and thickening, whereas the initiation of growth is not seen in TPS. Red arrows indicate the differential thickness of the cell walls of fibers from the two tissue types. Scale bars shown in the microscopic sections of TPS and MPS and in the figure of whole plant are 20 μm and 10 cm in length. [file 12870_2021_3252_MOESM4_ESM.docx]

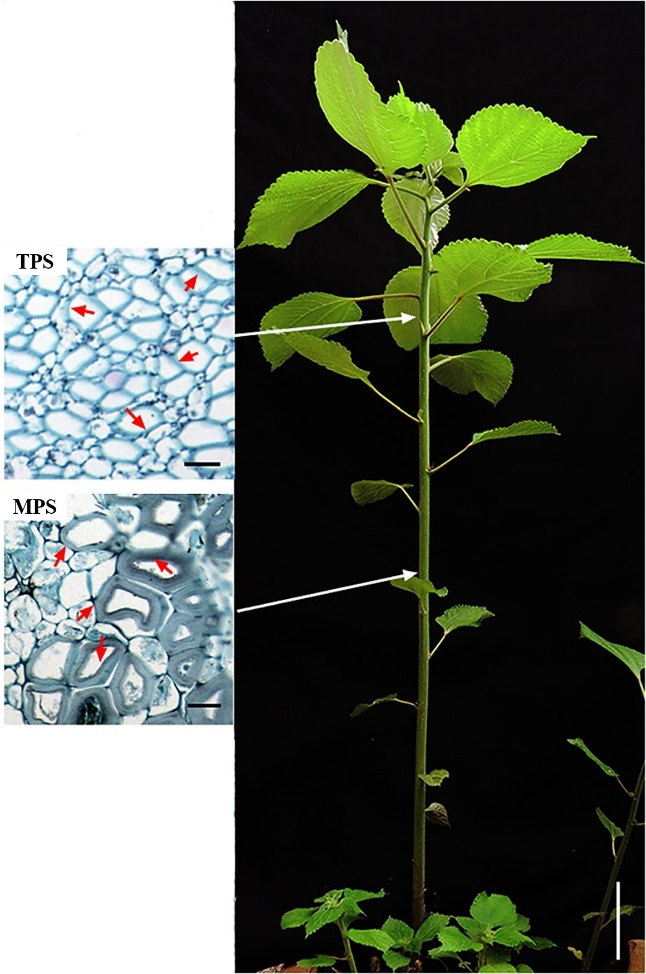


**Fig. S1** Microscopic observation of fiber cells from the stem barks of ramie (published by Chen et al. 2014, BMC Genomics, 15:919). Barks collected from the top (TPS) and middle (MPS) sections of ramie stems were used for phosphoproteome analysis in this study. MPS is characterized by secondary cellular wall growth initiation and thickening, whereas the initiation of growth is not seen in TPS. Red arrows indicate the differential thickness of the cell walls of fibers from the two tissue types. Scale bars shown in the microscopic sections of TPS and MPS and in the figure of whole plant are 20 μm and 10 cm in length.
