# Supplementary material for: Phosphoproteome analysis reveals an extensive phosphorylation of proteins associated with bast fiber growth in ramie
Source: BMC Plant Biol. 2021 Oct 16;21:473. doi: 10.1186/s12870-021-03252-7 (PMC8520194; doi:10.1186/s12870-021-03252-7)
Supplement: Supplementary file 5 — Additional file 5: Figure S2. Evaluation of the repeatability among replicate samples based on Pearson correlation analysis. The numbers in the graph indicate the correlation coefficient, and the red and blue table represent the positive and negative correlations, respectively. TPS and MPS indicate the phosphoproteome for the barks collected from the top and middle sections of stems, respectively. [file 12870_2021_3252_MOESM5_ESM.docx]

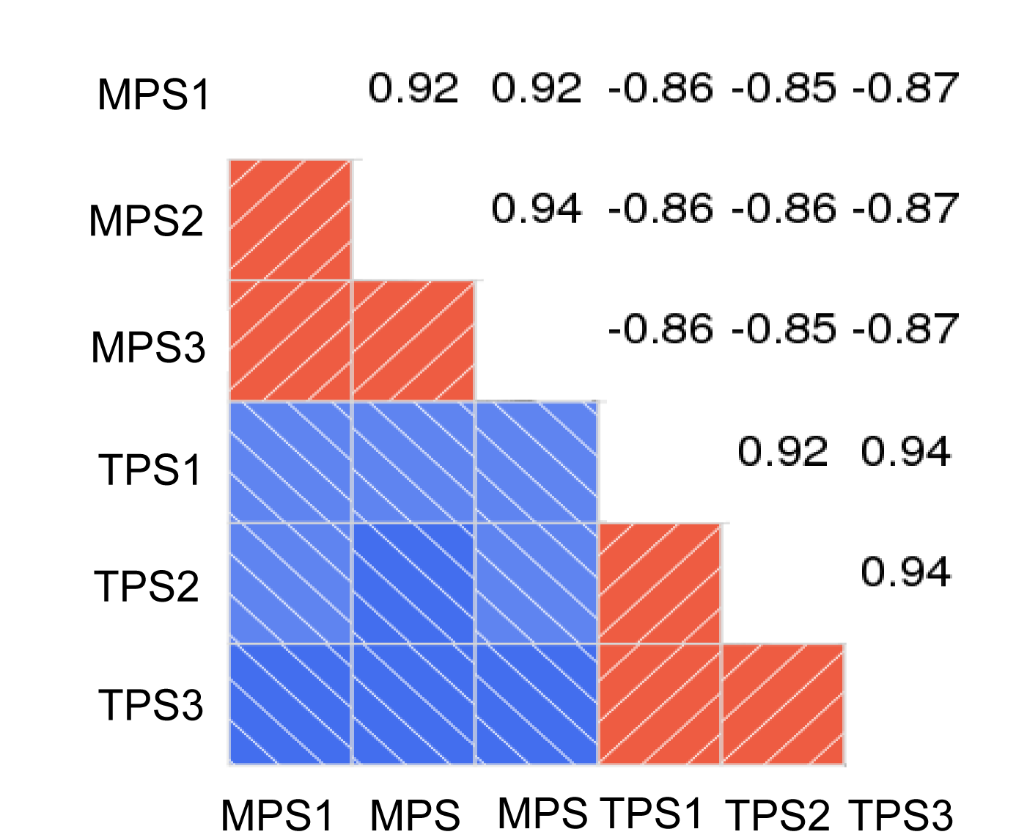


**Fig. S2** Evaluation of the repeatability among replicate samples based on Pearson correlation analysis. The numbers in the graph indicate the correlation coefficient, and the red and blue table represent the positive and negative correlations, respectively. TPS and MPS indicate the phosphoproteome for the barks collected from the top and middle sections of stems, respectively.
